# Convolutional Neural Networks for Recognition of Lymphoblast Cell Images

**DOI:** 10.1155/2019/7519603

**Published:** 2019-06-02

**Authors:** Tatdow Pansombut, Siripen Wikaisuksakul, Kittiya Khongkraphan, Aniruth Phon-on

**Affiliations:** Department of Mathematics and Computer Science, Faculty of Science and Technology, Prince of Songkla University, Pattani 94000, Thailand

## Abstract

This paper presents the recognition for WHO classification of acute lymphoblastic leukaemia (ALL) subtypes. The two ALL subtypes considered are T-lymphoblastic leukaemia (pre-T) and B-lymphoblastic leukaemia (pre-B). They exhibit various characteristics which make it difficult to distinguish between subtypes from their mature cells, lymphocytes. In a common approach, handcrafted features must be well designed for this complex domain-specific problem. With deep learning approach, handcrafted feature engineering can be eliminated because a deep learning method can automate this task through the multilayer architecture of a convolutional neural network (CNN). In this work, we implement a CNN classifier to explore the feasibility of deep learning approach to identify lymphocytes and ALL subtypes, and this approach is benchmarked against a dominant approach of support vector machines (SVMs) applying handcrafted feature engineering. Additionally, two traditional machine learning classifiers, multilayer perceptron (MLP), and random forest are also applied for the comparison. The experiments show that our CNN classifier delivers better performance to identify normal lymphocytes and pre-B cells. This shows a great potential for image classification with no requirement of multiple preprocessing steps from feature engineering.

## 1. Introduction

Acute lymphoblastic leukaemia (ALL) is an acute malignancy of white blood cells, causing over production of immature lymphocytes, known as lymphoblasts, in the bone marrow. The disease progresses rapidly and inhibits the production of normal cells causing death among children and young adults. ALL is a heterogeneous disease, meaning that distinct treatments are required for different groups of patients according to subtypes of the leukaemia. Individual ALL subtypes response differently to particular chemotherapy. Therefore, subtype recognition provides essential prognostic information for a treatment planning.

Considering WHO classification, ALL subtypes can be subdivided as T-lymphoblastic leukaemia (pre-T), B-lymphoblastic leukaemia (pre-B), and mature-B lymphoblastic leukaemia (mature-B) [1]. The identification of the subtypes requires a multiparametric approach, including morphology, immunophenotype, cytogenetic, and molecular findings. Despite having advanced techniques, a morphological examination of blood smear samples is still a procedure for initial screening. The morphological examination can be assisted by computer-based systems. There have been growing interests in developing tools using image analysis and pattern recognition methods for quantification and identification of leukocytes [2–5]. They could bring the efficacy to the analysis in terms of time and accuracy and assist pathologists in studying different patterns or cells from microscopic images. Since this system requires only images not blood samples, it offers low-cost methods and enables historical data records for future used in remote diagnostic systems.

The morphology of lymphocytes and ALL subtypes exhibits large variation among cells in the same class. At the same time, they show many characteristics which resemble cells belonging to different families. Some examples are shown in Table 1 which presents five samples of blood microscopic images of normal lymphocytes acquired from Labati et al. [6] and pre-T and pre-B lymphoblasts from the American Society of Hematology (ASH) image bank [7]. Morphologically, lymphocytes present a compact nucleus with smooth boundary, blue-purple nucleus color, and low nucleus/cytoplasm (N/C) ratios [6, 8]. Instead, lymphoblasts exhibit irregularities with rough nucleus boundary, sparse red-purple nucleus color, and high N/C ratios [6, 8]. Considering ALL subtypes based on WHO classification, pre-T and pre-B lymphoblasts have the following characteristics.

Pre-T cells vary considerably from small blasts with very condensed nuclear chromatin and indistinct nucleoli to larger blasts with finely dispersed chromatin and prominent nucleoli [1]. The sparse amount of cytoplasm is commonly presented. Some cytoplasmic granulation is frequently found, look like grains of dust in most cases and occasionally exhibits visible large granules [9]. Nuclei range from round to irregular to convoluted. Some characteristics, such as cleaved nuclei, cytoplasmic protrusion, or hand-mirror form, are also presented [7].

Pre-B cells exhibit various characteristics from small-sized cell with scant cytoplasm, condensed nuclear chromatin, and inconspicuous nucleoli to medium-sized cell with moderate amounts of light blue cytoplasm, occasionally, finely dispersed nuclear chromatin, and relatively prominent nucleoli [1]. Most have a high N/C ratio. Other characteristics, such as elongated form, hand-mirror form, round or irregular nuclear contour, are occasionally presented [7].

The automated cell recognition of these subtypes has to handle this complex problem. A dominant approach is hand-crafted feature engineering with classification algorithms such as SVM, kNN, and MLP. For this approach, features are first extracted using image processing techniques and domain knowledge, and the combination of useful features is selected as input for the classification algorithms. This approach has some disadvantages, consequence of hand-crafted feature engineering. First, the approach may require domain knowledge expertise in determining useful features. Second, it relies on image processing techniques in extracting useful features without introducing additional bias and error. Third, feature extraction operations are difficult to automate and possibly time-consuming.

Our deep learning approach implements a convolutional neural network which directly takes in pixel's values from images and slowly constructs useful features through the use of multilayer architecture. These features are then used to recognize the patterns relevant to the classification problem. Another consideration is the size of data set. The number of data is limited in many real-world problems, as also shown in our problem. From this requirement, we utilize appropriate data augmentation techniques to increase the number of input images for training.

Our study aims to apply a deep learning approach for developing a recognition of lymphocytes and ALL subtypes including pre-T and pre-B cells from blood microscopic images. We omit mature-B since it is in rare cases compared to the other two subtypes. To assess the performance of our deep learning approach, we compare the prediction accuracy and sensitivity of our CNN classifier with SVM classifier employing hand-crafted feature engineering. To ensure a fair comparison, the SVM classifier is enhanced with feature selection and GA-based parameters optimization. In addition, two traditional machine learning classifiers, MLP and random forest, are also considered to realize where our CNN approach can be situated among other machine learning methods.

## 2. Related Work

The analysis of hematological images is generally divided into four major steps consisting of image preprocessing, segmentation, feature extraction and selection, and classification. A considerable amount of works has been focused on leukocytes segmentation [10–16]. For example, Mohapatra et al. [14] have proposed the segmentation method using color-based clustering to obtain nucleus region and cytoplasm area from stained blood smear images. SVM classifiers are applied with relevant features and gain satisfactory results.

The automated classification of different types of white blood cells has been demonstrated in [17, 18]. In [17], Osowski and Markiewicz have presented fully automatic system able to recognize 17 classes of myelogenous leukaemia from images of bone marrow aspirate. Cells are segmented using watershed algorithm combined with region-growing and edge detection techniques. 117 descriptive features have been generated and selected using linear SVM. This algorithm has been improved by Osowski et al. [18]. The latter work has presented feature selection using genetic algorithms for feature selection along with SVM learning algorithm. The algorithm increases accuracy of the recognition by more than 25%.

Reta et al. [19] have proposed the method to categorize the two types of leukaemia, ALL and acute myeloid leukaemia (AML). The segmentation of blood cells is performed using contextual color and texture information to identify nucleus and cytoplasm region as well as to separate overlapped blood cells. The morphological, statistical, texture, size ratio, and eigen values features are extracted after segmentation to be used by various machine learning classifiers available in Weka.

Recently, deep learning techniques become promising choices for medical image analysis. For example, works in [20–22], convolutional neural networks have been applied as a methodology in microscopic analysis. Song et al. [20] have used deep learning method based on a superpixel and convolutional neural network to detect the cytoplasm region in cervical cancer cell segmentation. The CNN approach is compared to different algorithms which are backward propagation neural network (BPNN), probabilistic neural networks (PNN), support vector machine (SVM), and learning vector quantization (LVQ) algorithms. CNN is superior to other algorithms and produces an accuracy of 94.50% for nucleus region detection. For cytoplasmic and nucleus segmentation, CNN outperforms all three state-of-the-art methods as measured by F-measure, precision, and recall.

Zhao et al. [21] have proposed an automatic detection of white blood cells (WBCs) from peripheral blood images and classification of five types of WBCs: eosinophil, basophil, neutrophil, monocyte, and lymphocyte. Eosinophil and basophil from other WBCs are first classified by SVM with a granularity feature. Other three types are then recognized using convolutional neural network to extract features, and random forest uses these features to classify those WBCs.

Litjens et al. [22] have introduced deep learning as a technique to improve the objectivity and efficiency of histopathologic slide analysis. Convolutional neural networks are trained in two experiments which are prostate cancer identification in biopsy specimens and breast cancer metastasis detection in sentinel lymph nodes. They show that this system holds great promise to reduce the workload of pathologists with increasing objectivity of diagnoses.

## 3. Materials and Methods

Blood microscopic images are acquired from two different collections. The first collection comprises normal white blood cells obtained from Labati et al. [6]. We acquire 93 color images, each containing single normal WBC. The second collection is composed of ALL subtypes: pre-T and pre-B cells from ASH image bank [7]. In the entire blood smear images, a single pre-T or pre-B cell is manually cropped from the whole scene. Each image contains a single cell and is rescaled to equal size of 256 × 256 pixels. In the conventional approaches, we need to define region of interest (ROI) from the background. Manual segmentation is performed to mask the whole cell region and the nucleus area. Then, the masks of nucleus and cytoplasm can be defined and stored as contour labels of the object.

In this work, a convolutional neural network applied for an image classification problem is called ConVNet. The ConVNet method directly uses RGB values of the cell images for the learning procedure which automatically extracts image features through a multilayer architecture. For the dominant approach, feature values are extracted from the object information of the image using image processing techniques. We conduct these values into the implementation of SVM with GA-based feature selection and parameters optimization, namely, SVM-GA. These same feature values are also employed by the standard approaches of MLP and random forest. Further details of the proposed approach and implementation of the classifiers used in this work are presented in the following sections.

## 4. Classification of ALL Using ConVNet

### 4.1. Convolutional Neural Networks

Convolutional neural networks have shown success in image classification [23–25]. The strength of a CNN lies on its ability to employ a multilayer architecture to automatically extract high-level features through a series of convolutional, nonlinear transformation, downsampling (pooling), and fully connected layers of the network.

To train a CNN for image classification, first the network architecture must be designed. This task is to determine the types, number, and order of layers in the network. The designed network, given a set of 2D images along with their corresponding class labels, attempts to find features useful for distinguishing the classes. A CNN employs a learning method that consists of two repeated and alternated passes, naming feedforward and backward pass.

A typical CNN's feedforward pass performs two major tasks. The first task is feature extraction via the use of multiple convolutional feature extraction (CFE) layers. For this task, an image is passed through multiple CFE layers in a serial manner. A CFE layer consists of three sublayers: a convolutional sublayer, followed by a nonlinear transformation sublayer, and then by a pooling sublayer. Each CFE layer takes features from the previous layer and constructs higher-level features. This process often repeats many times in order to eventually extract high-level features from the image. These features then become input for the fully connected layers in the second task of a feedforward pass, which performs classification of the input image and obtains some error.

In a backward pass, the error obtained from a feedforward pass propagates backward to adjust the weights in the convolutional sublayers, and therefore, they can better extract features relevant to the classification problem. The same error is also used to find proper weights for the fully connected layers.

### 4.2. Architecture of ConVNet

**Figure 1:**
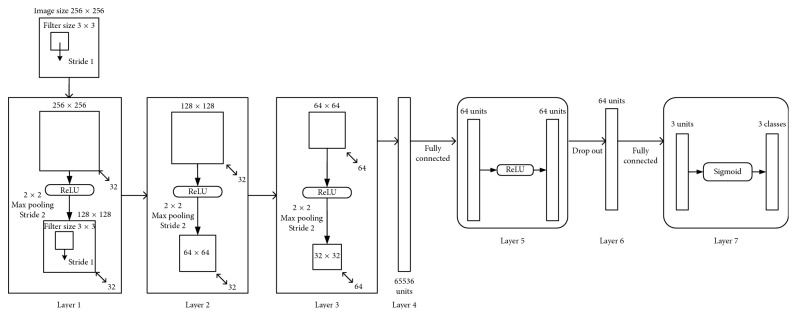
Architecture of ConVNet. The CNN consists of seven layers. Layers 1, 2, and 3 implement feature extraction of cell images. Layer 4 transforms 64 extracted features into one-dimensional array of size 65536. Layer 5 maps 65536 inputs into 64 outputs. Layer 6 drops 50 percent of the 64 inputs at random. Layer 7 performs classification of 3 types of ALL subtypes.

The overall architecture of ConVNet used in this study is shown in Figure 1. The network consists of seven layers, excluding the input layer. The input layer takes in a 256 × 256 RGB color image when each color channel is processed separately.

The first, second, and third layers of ConVNet are CFE layers. The first and second CFE layer each applies 32 of 3 × 3 filters to an image in the convolutional sublayer. The image's border is padded with 0 to maintain the image size of 256. The nonlinear transformation sublayer employs the ReLU activation function. The max pooling sublayer applies a 2 × 2 filter to the image which results in reducing the image size to its half. The third CFE layer has similar structure to the first one, except the number of filters is 64. At this point, ConVNet extracts 64 features, each represented by a 32 × 32 array for each color channel.

The fourth layer is a flatten layer. The flatten layer transforms a multidimensional array into one-dimensional array by simply concatenating the entries of the multidimensional array together. The output of this flatten layer is a one-dimensional array of size 65536. The fifth layer is a fully connected artificial neural network (ANN) with the ReLU activation function that maps 65536 input values to 64 output values. The sixth layer is a dropout layer. 50 percent of the input values coming into this layer are dropped to zero to reduce the problem of overfitting. The seventh layer is a fully connected ANN with the sigmoid activation function that maps 64 input values to 3 class labels.

### 4.3. Procedure of ConVNet

**Figure 2:**
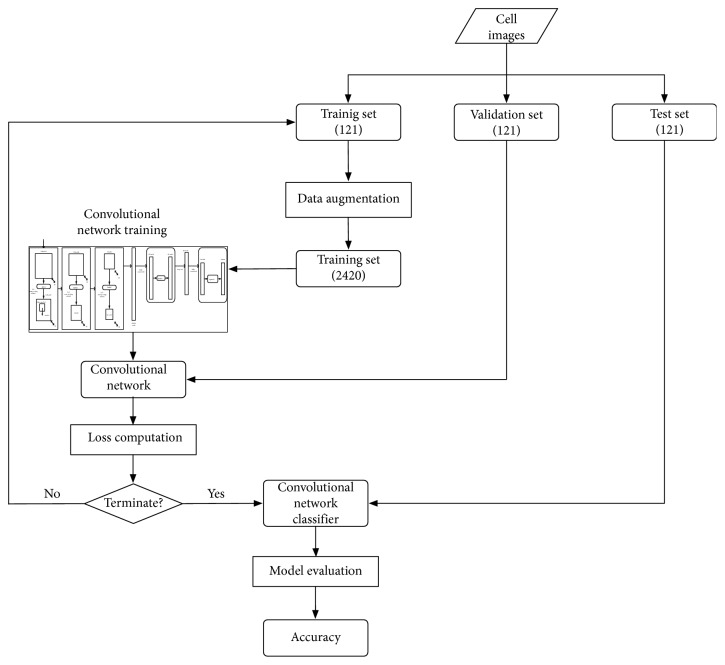
Image classification using ConVNet.

The overall procedure of image classification using ConVNet is presented in Figure 2. Since a large amount of data is essential in achieving high performance for CNN, we utilize data augmentation techniques to increase the number of images in the training set from 121 to 2420 images. The operations used for data augmentation are horizontal flip, shearing within 0.2 radians in the counterclockwise direction and zooming between 0.8 and 1.2.

First, we train ConVNet using the data in training set to find appropriated filters' weights in the three convolutional sublayers and the weights that yield minimum error in the two fully connected layers. Next, we evaluate ConVNet using the data in the validation set to obtain validation error and cross-entropy loss. We then train ConVNet again using a new training set created from data augmentation of the original 121 training images. We repeat the training of ConVNet in this same procedure until we complete 50 epochs. Last, we evaluate the performance of ConVNet using data in the test set.

### 4.4. Time-Complexity of ConVNet

The time-complexity of ConVNet includes the time costs for the three CFE layers: the flatten layer, the dropout layer, and the two fully connected layers. For both training and testing, the time costs for the CFE layers dominate the overall complexity. Furthermore, for each CFE layer, the time cost for a convolutional sublayer succeeds the time cost for a nonlinear transformation and a max pooling sublayers combined.

In general, the total time-complexity of all convolutional sublayers can be written as follows [26]:

(1) $$\begin{array}{c} O\left(\overset{d}{\underset{l=1}{\sum }}n_{l-1}\cdot s_{l}^{2}\cdot n_{l}\cdot m_{l}^{2}\right), \end{array}$$

where *l* is the index of a convolutional sublayer, *d* is the number of convolutional sublayers, *n*_*l*_ is the number of filters in the *l*th layer, *s*_*l*_ is the spatial size (length) of the filter in the *l*th layer, and *m*_*l*_ is the spatial size of the output feature map in the *l*th layer.

For ConVNet, which consists of three convolutional sublayers, the time-complexity can be estimated by calculating the total number of convolutional operations performed (per image) in a single feedforward or backward pass (as shown in Table 2).

In terms of the difference between training and testing times per image, training takes three times as long as testing since it requires both feedforward and backward passes while testing only performs a feedforward pass [26]. The classification step operates in the same manner as testing; therefore, it includes only the time for one feedforward pass.

## 5. Feature Extraction for SVM-GA, MLP, and Random Forest

To facilitate the process of cell recognition, we need numerical feature values imitating the details of characteristics presenting best correlated within the same class and enhancing the differences for cell images belonging to different classes. These detailed values can be used to detect variations in shape, cell size, granulation, intensity, color, etc. The segmented nucleus and cytoplasm of each individual cell image are described by various numerical values representing features from three main groups: geometrical, textural, and color features. All features are presented in Table 3. The 46 features are generated for classification and summarized as follows.

**Figure 3:**
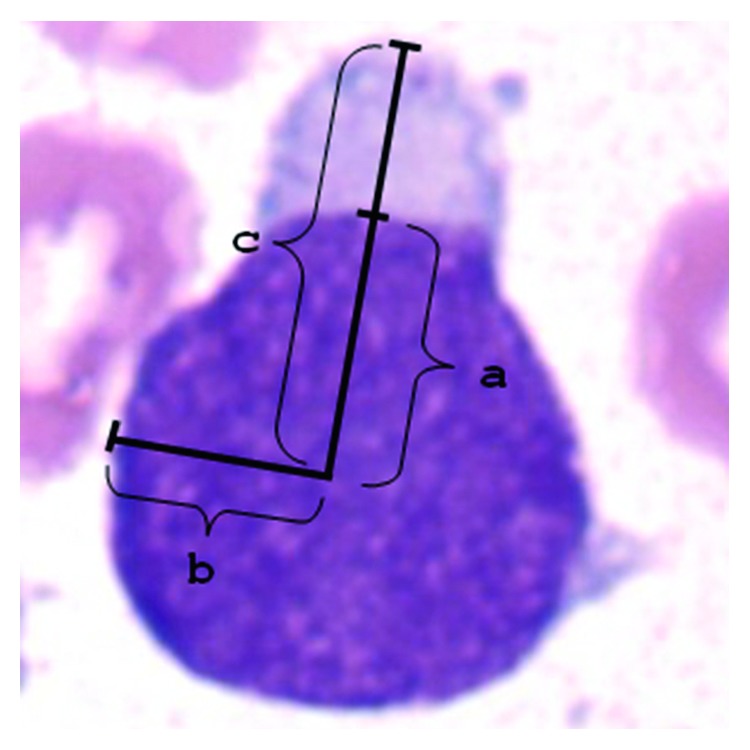
Identification of hand-mirror morphology measured by the proportion *a*+*c*/*b*+*c*.

- The geometrical feature is used to describe the differences of the structure, shape, and size of leukocyte as geometry. The geometrical feature extraction is mainly based on a region-based and a contour-based approach. In the region-based approach, each cell image is first converted to a binary image, and then, the features 1–7 are extracted from cell geometry. Features 1–5 are adopted from Mohapatra et al. [8]. Features 6 and 7 are newly presented in this work, and they can be defined as follows.
- Feature 6 is the measurement of symmetry by folding a nucleus shape with respect to a line of symmetry referred to the nucleus major axis. The numerical value of shape symmetry can be defined as

(2) $$\begin{array}{c} \text{shape symmetry}=\;\;\frac{{\text{part}}_{1}}{{\text{part}}_{2}}, \end{array}$$

  where part_1_ is the overlapping area between two separated parts with respect to the line of symmetry and part_2_ is the largest area between two separated parts. Therefore, the nucleus shape is more symmetry when the value is closer to 1.
  Feature 7 is to measure how a cellular presents the hand-mirror shape. Hand-mirror cell (HMC) lymphoid leukaemia is an unusual variant of ALL, in which the lymphoblasts manifest distinctive hand-mirror morphologic features. As shown in Figure 3, the proportion *a*+*c*/*b*+*c* is used to identify how the cell is close to a pre-B cell or pre-T cell. The distances *a* and *b* are the semimajor axis and the semiminor axis of nucleus, respectively. *c* is the maximum distance from the center of the nucleus to the hand-mirror part of the cell.

  Features 8–15 are based on a contour-based approach where the nucleus and cellular boundary roughness are measured using two quantitative methods: fractal geometry and contour characteristics. These features are adopted from Mohapatra et al. [8].
(ii) Textural features provide essential properties that reflect the organization of the cellular surface. Nuclear chromatin patterns, granulation, and cell smoothness are essential diagnostic descriptors to differentiate cells among different types. Each cell image is first converted to a grayscale image. Thereafter, the methods based on the Haar wavelet transformation [27] in features 16–21, Haralick's texture [28] in features 22–26, and Fourier descriptors [29] in features 27–34 are applied to detect the textural transformations. The Fourier descriptors are obtained from the two-dimensional discrete forward and inverse Fourier transforms in features 27–30 and 31–34, respectively.
(iii) The color appearance is an important characteristic that is used to examine the abnormality of lymphocytes since normal and malignant cells have different staining capacity and granulation. Excessive staining capacity of nuclei normally appears in chromatin abnormality, and variation in color intensity usually presents due to the existence of granules. The color variation can be measured as mean color intensity in RGB and HSV color space. These features are calculated from nucleus region (35–40) and cytoplasm (41–46).

## 6. Classification of ALL Using SVM with GA-Based Parameters Optimization

### 6.1. Support Vector Machines for Classification

Support vector machines (SVMs) are based on the concept of decision planes that define decision boundaries and perform classification tasks by constructing hyperplanes in a multidimensional space [30]. To construct an optimal hyperplane, SVM employs an iterative training algorithm which is used to minimize an error function described in the following equation:

(3) $$\begin{array}{c} \frac{1}{2}w^{T}w+C\overset{N}{\underset{i=1}{\sum }}\varepsilon_{i}, \end{array}$$

subject to the constraints *y*_*i*_(*w* ^*T*^ *φ*(*x*_*i*_)+*b*) ≥ 1 − *ε*_*i*_ and *ε*_*i*_ ≥ 0, *i*=1,2,…, *N*, where *C* is the penalty parameter, *w* is the vector of coefficients, *b* is a constant, and *ε*_*i*_ represents the parameter for handling input data *i*. The index *i* labels the *N* training cases, *y*_*i*_ ∈ {−1,1} represents the class label, and *x*_*i*_ represents the independent variable. The kernel *φ* is used to transform data from the input space to the feature space.

One important choice when using SVMs is the selection of an appropriate kernel function that is needed for efficiently handling nonlinearly separable data sets. The radial basis function (RBF) kernel is often chosen for this purpose [31, 32], but it has a drawback that all input features are considered equally important when computing similarities between two feature vectors. Therefore, to make optimal use of SVMs with RBF kernels, preprocessing of the input features is important when one wants to achieve the highest possible accuracy. The RBF kernel on two samples *x*_*i*_ and *x*_*j*_ is defined in the following equation:

(4) $$\begin{array}{c} K\left(x_{i},x_{j}\right)\approx \varphi {\left(x_{i}\right)}^{T}\varphi \left(x_{j}\right)=\exp\left(-\gamma {\left\|x-x^{\prime }\right\|}^{2}\right), \end{array}$$

where *γ* is the gamma parameter.

The behavior of the model depends on both parameters *C* and *γ*. The parameter *C* actually determines how much penalty should be given for misclassification. The parameter *γ* can be seen as the inverse of the radius of influence of samples selected by the model as support vectors. As the *γ* increases, the support vector has less wide-spread influence which makes the algorithm try harder to avoid misclassifying training data and leads to overfitting.

### 6.2. GA-Based Feature Selection and Parameters Optimization

**Figure 4:**
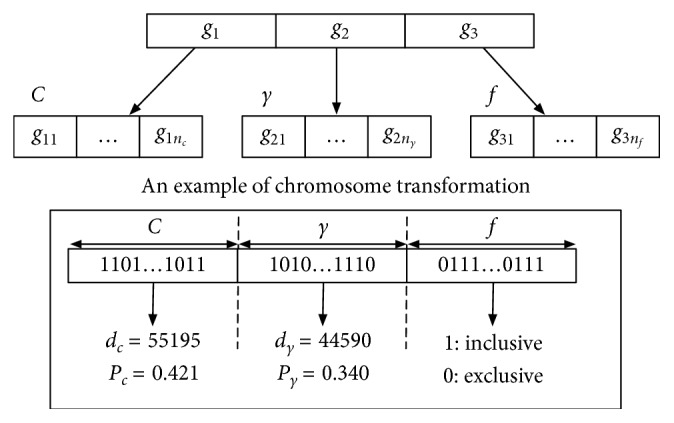
The locus of chromosome consists of three parts: *C*, *γ*, and the features mask *f*.

From the aforementioned considerations of feature selection and parameter tuning, we adopt the GA approach from Huang and Wang [33] for these optimization tasks. For the chromosome encoding in this work, a string of binary values is used to define three parts: *C*, *γ*, and the feature mask *f*. In Figure 4, *g*_1_, *g*_2_, and *g*_3_ define bit strings of *C*, *γ*, and *f*, respectively. The lengths of each part are *n*_*C*_, *n*_*γ*_, and *n*_*f*_, which have the number of bits depending on the size of parameters used by the kernel function and the number of features from data set. *C* and *γ* parts in the bit strings in Figure 4 must be decoded from binary to decimal by the following equation[33]:

(5) $$\begin{array}{c} P=P_{\text{min}}+\frac{P_{\text{max}}-P_{\text{min}}}{2^{l}-1}\times d, \end{array}$$

where *P*_max_ and *P*_min_ are the maximum and the minimum values of the parameter, *d* is decimal value of bit string, and *l* is the length of bit string.

The GA operators used in our approach are reproduction or selection by roulette wheel mechanism, single-point crossover, and mutation using bit alteration. Two parents are first selected by selection operator for reproduction. Based on a random probability of crossover *p*_c_, if crossover occurs, a position *x* of the string with size *l* is randomly chosen, and alleles at position *x* to *l* are exchanged from one parent to the other. If no crossover occurs, the parents are directly copied to the new population. Different from Huang and Wang [33], we consider the string of the parameters (*C* and *γ*) part and the feature mask part differently since they have different meanings. The first part will be converted to decimal value, and the second part of the feature mask will be used directly. Therefore, the crossover operator performs for each part separately.

Apart from the crossover operator, the mutation operator is used to perturb bit value with a low probability to maintain genetic diversity. Each bit of an individual can be reversed from 0 to 1 or 1 to 0 with probability *p*_*m*_. This applies for all individuals which are placed in the new population.

**Figure 5:**
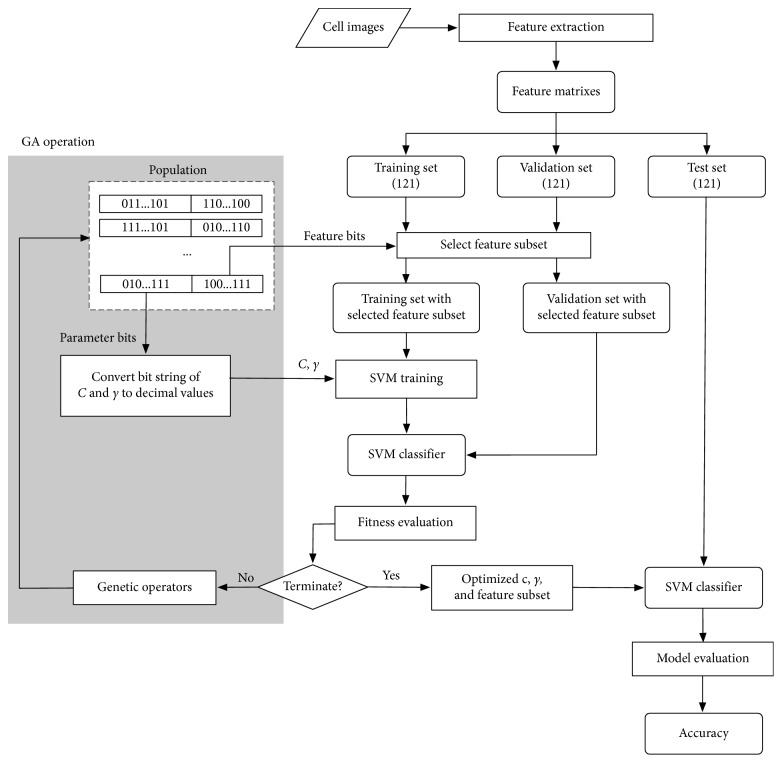
Feature selection and parameters optimization using the GA-based technique from [33].

The overall procedure of the SVM classifiers with GA-based feature selection and parameters optimization is presented in Figure 5. In the SVM learning, GA operations are used to adjust proper parameters. After the trained SVM classifier is obtained in each round, the validation data with selected feature subset and parameters are tested by the SVM classifier. Each chromosome is evaluated according to the average classification accuracy obtained from the validation data. The optimized parameters (*C* and *γ*) and the feature subset are finally obtained for the final SVM classifier which is evaluated using data in the test set.

## 7. Experiments

The original data set contains 363 images. There are two types of the data set, one is the cell images for ConVNet and the other is the feature values extracted from the corresponding cell images for SVM-GA, MLP, and random forest. Both types of data are divided into training, validation, and testing data. Each set contains 31 normal cell images, 45 pre-T cell images, and 45 pre-B cell images. Samples from the data set are randomly selected with ten different seeds to generate ten different combinations for both types of data. Training and validation data are used for building the models and testing data are for evaluating the classifiers' performance.

The quality of models is evaluated by accuracy and sensitivity. With *n* number of classes, there is a confusion matrix consisting of the elements *C*_*ij*_. The diagonal entries *C*_*ii*_ represent the numbers of correctly recognized classes. Typically, the accuracy *A* can be defined in the following equation:

(6) $$\begin{array}{c} A=\frac{\sum_{i=1}^{n}C_{ii}}{\sum_{i=1}^{n}\sum_{i=1}^{n}C_{ij}}. \end{array}$$

Sensitivity *S*_*i*_ for class *i* measures the ratio of the number of patterns that are correctly recognized in class *i* to the total number of patterns in class *i*, defined in the following equation [34]:

(7) $$\begin{array}{c} S_{i}=\frac{C_{ii}}{\sum_{j=1}^{n}C_{ij}}. \end{array}$$

Parameter settings for all four approaches are summarized in Table 4.

## 8. Results and Discussion

In the SVM-GA approach, the first task is to obtain an SVM classifier for a binary classification of normal lymphocytes and lymphoblast cells. The second SVM model is produced to distinguish pre-T and pre-B cells. Therefore, the SVM models with the selected feature subsets and the optimized parameters are used to classify three classes of cells: normal lymphocytes, pre-T, and pre-B cells. If the testing data are identified as normal, then the result is obtained. Otherwise, the testing data are further identified if it is pre-T or pre-B cells.

To evaluate the performance of our deep learning approach, we compare ConVNet with the dominant approach of SVM-GA and two traditional machine learning methods, namely, MLP and random forest.  Table 5 depicts the accuracy results obtained from these approaches taking ten test sets and shows the average with standard deviation over the ten performance estimates. Considering the average accuracy, the two traditional approaches cannot achieve the accuracy above 80% while ConVNet and SVM-GA yield the average accuracy above 80% and produce comparable results with the difference on a very small margin. From the ten set runs, most of the results obtained by both ConVNet and SVM-GA are above 80% and have the number approximately ranging from 78–86%.

To explore the sensitivity according to each class, Table 6 depicts the comparative results of the sensitivity according to each class over ten test sets between ConVNet and SVM-GA, and Table 7 displays the comparative sensitivity results between MLP and random forest. Starting from the identification of normal lymphocytes, ConVNet, MLP, and random forest provide comparable high average sensitivity of almost 100%. These three methods clearly outperform SVM-GA which can only achieve less than 95% sensitivity in identifying normal lymphocytes.

Comparing two subtypes, ConVnet, MLP, and random forest produce similar results in identifying pre-T cells with 68–70% sensitivity; however, SVM-GA is able to obtain higher sensitivity of 75%. For the classification of pre-B cells, ConVNet and SVM-GA deliver the sensitivity above 80% which is higher than those from MLP and random forest.

Considering the variances in the sensitivity measurements for all approaches, ConVNet produces lower standard deviations for lymphocyte and pre-B classifications. MLP and random forest generate high variances over ten test runs for pre-T and pre-B identifications whereas SVM-GA produces the highest variances among all four approaches for all three types of cell image classification. This behavior may be the indication of overfitting.

The final results are drawn from the confusion matrix from the two best classifiers, ConVNet and SVM-GA, to reveal how the classifiers identify the testing data. The matrixes are chosen from the worst and best results of the accuracy depicted in Tables 8 and 9, respectively. The results show the relative misclassification between pre-T and pre-B cells. This is due to the high similarity of these two classes.

## 9. Conclusions

In this work, we present a deep learning approach to recognize normal lymphocytes and ALL subtypes defined by WHO classification. We implement a CNN, namely, ConVNet, which directly takes raw images and automatically discovers useful features through a series of multilayer architecture. The performance of our deep learning model is evaluated against a dominant approach of SVM classifier, namely, SVM-GA, and two traditional machine learning approaches including MLP and random forest.

In terms of prediction accuracy, the deep learning approach of ConVNet and the dominant approach of SVM-GA are able to clearly outperform the two traditional approaches of MLP and random forest. In fact, the average accuracy of ConVNet and SVM-GA is comparable. However, when the sensitivity which measures the accuracy according to a particular class has been explored, we observe a clearer picture of the two classifiers' performance. ConVNet performs better in detecting normal lymphocytes and slightly better in detecting pre-B cells. Regarding the classification of pre-T cells, neither classifiers can deliver good results above 80% accuracy. Although SVM-GA demonstrates its ability to detect pre-T cells better than ConVNet, it may have suffered from overfitting, as suggested by its consistently high variances.

For the problem of recognizing lymphoblast cells, a deep learning approach of CNN is superior to MLP and random forest in all three classes, and it is able to outperform the dominant approach of SVM classifier employing GA-based parameters optimization for two out of the three classes. Taking into consideration that a CNN method requires no hand-crafted feature engineering, which is an error-prone and possibly time-consuming preprocessing step, this deep learning approach demonstrates a great potential for lymphoblast cell image classification.

## Figures and Tables

**Table 1:** Sample images of the considered white blood cells: lymmphocyte, pre-T, and pre-B lymphoblasts.

Lymphocyte	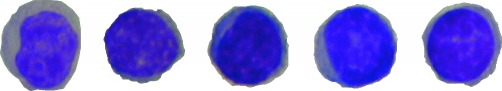
Pre-B	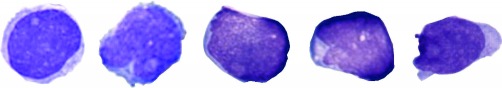
Pre-T	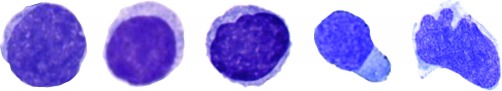

**Table 2:** ConVNet's total convolutional operations.

*l*	*n*_*l*−1_	*s*_*l*_	*n*_*l*_	*m*_*l*_	Number of convolutional operations in the *l*th layer
1	1	3	32	256	1 × 3^2^ × 32 × 256^2^=18,874,368
2	32	3	32	128	32 × 3^2^ × 32 × 128^2^=150,994,944
3	32	3	64	64	32 × 3^2^ × 64 × 64^2^=75,497,472
Total convolutional operations					245,366,784

**Table 3:** Summary of features extracted using image processing.

No.	Feature	ROI	Type	Description
1	N/C ratio	Cellular	Geometric	Ratio of number of pixels in nucleus to those in cytoplasm
2	Form factor	Nucleus		Ratio of number of pixels in nucleus to its perimeter
3	Roundness	Nucleus		Measurement of how nucleus shape is close to a circle
4	Eccentricity	Nucleus		Ratio of major axis to minor axis
5	Compactness	Nucleus		Degree to which a shape is compact
6	Symmetry	Nucleus		Ratio between two parts around the nucleus major axis
7	Hand-mirror	Cellular		Measurement of how the hand-mirror part of the cell forms
8	Fractal geometry	Nucleus		Degree to which the nucleus boundary is irregular by calculating Hausdorff dimension
9–11	Contour	Nucleus		Variance, skewness, and kurtosis of distances between centroid and contour points along the nucleus boundary
12	Fractal geometry	Cellular		Degree to which the cellular boundary is irregular by calculating Hausdorff dimension
13–15	Contour	Cellular		Variance, skewness, and kurtosis of distances between centroid and contour points along the cellular boundary

16–18	Haar wavelet	Nucleus	Texture	Mean of *A*_*n*_, *H*_*n*_, and *V*_*n*_
19–21	Haar wavelet	Nucleus		Variance of *A*_*n*_, *H*_*n*_, and *V*_*n*_
22–26	Haralick	Nucleus		Contrast, correlation, homogeneity, energy, and entropy of Haralick's texture feature values
27–34	Fourier descriptors	Nucleus		Mean, standard deviation, skewness, and kurtosis of the frequency components obtained from discrete forward (27–30) and inverse (31–34) Fourier transforms

35–37	Color in RGB	Nucleus	Color	Mean color intensity of red, green, and blue in a nucleus area
38–40	Color in HSV	Nucleus		Mean color intensity of hue, saturation, and value in a nucleus area
41–43	Color in RGB	Cytoplasm		Mean color intensity of red, green, and blue in a cytoplasm area
44–46	Color in HSV	Cytoplasm		Mean color intensity of hue, saturation, and value in a cytoplasm area

**Table 4:** Summary of parameter settings for the ConVNet, SVM-GA, MLP, and random forest.

Methods	Parameters	Setting
ConVNet	Filter size (convolutional layer)	3 × 3
	Filter size (max pooling layer)	2 × 2
	Batch size	121
	Epoch	50
	Learning rate	0.001

SVM-GA	Population size	100
	Number of generations	100
	Probability of crossover	0.80
	Probability of mutation	0.06
	Rate of elitism	0.05
	*n*_*C*_, *n*_*γ*_	20
	*n*_*f*_	42

MLP	Number of hidden layers	1
	Number of neurons in hidden layer	69
	Activation function	Logistic
	Batch size	121
	Epoch	10000
	Learning rate	0.001
	Momentum	0.7

Random forest	Number of classifiers	100
	Maximum depth	2

**Table 5:** Accuracy of ConVNet, SVM-GA, MLP, and random forest to identify lymphocytes, pre-T, and pre-B cells over ten test sets.

Test set	ConVNet	SVM-GA	MLP	Random forest
1	82.64	81.82	74.38	78.51
2	80.17	80.99	75.21	72.73
3	85.12	80.17	75.21	79.34
4	80.17	80.17	76.86	80.99
5	78.51	79.34	76.03	78.51
6	78.51	81.82	73.55	76.86
7	83.47	80.99	78.51	79.34
8	85.95	81.82	81.82	80.99
9	83.47	86.78	76.03	79.34
10	79.34	82.64	73.55	77.69
Average	81.74 ± 2.74	81.65 ± 2.05	76.12 ± 2.51	78.43 ± 2.38

**Table 6:** Sensitivity of ConVNet and SVM-GA to identify ALL subtypes over ten test sets.

Test set	ConVNet			SVM-GA		
	Lymphocyte	Pre-T	Pre-B	Lymphocyte	Pre-T	Pre-B
1	100.00	71.11	82.22	100.00	77.78	73.33
2	100.00	66.67	80.00	93.55	80.00	73.33
3	100.00	75.56	84.44	93.55	66.67	84.44
4	100.00	64.44	82.22	100.00	77.78	73.33
5	100.00	62.22	80.00	90.32	60.00	91.11
6	100.00	57.78	84.44	83.87	80.00	82.22
7	100.00	80.00	75.56	96.77	75.56	75.56
8	100.00	82.22	80.00	90.32	77.78	80.00
9	96.77	71.43	80.00	96.77	84.44	82.22
10	100.00	57.78	86.67	100.00	68.89	84.44
Average	99.68 ± 1.02	68.92 ± 8.65	81.56 ± 3.15	94.52 ± 5.28	74.89 ± 7.41	80.00 ± 6.02

**Table 7:** Sensitivity of MLP and random forest to identify ALL subtypes over ten test sets.

Test set	MLP			Random forest		
	Lymphocyte	Pre-T	Pre-B	Lymphocyte	Pre-T	Pre-B
1	100.00	68.89	62.22	100.00	73.33	68.89
2	100.00	68.89	64.44	100.00	64.44	62.22
3	100.00	62.22	71.11	100.00	64.44	80.00
4	100.00	68.89	68.89	100.00	71.11	77.78
5	96.77	64.44	73.33	100.00	62.22	80.00
6	100.00	64.44	64.44	100.00	71.11	66.67
7	96.77	77.78	66.67	100.00	73.33	71.11
8	100.00	80.00	71.11	96.77	80.00	71.11
9	100.00	75.56	60.00	100.00	75.56	68.89
10	100.00	57.78	71.11	96.77	66.67	75.56
Average	99.35 ± 1.36	68.89 ± 7.10	67.33 ± 4.45	99.35 ± 1.36	70.22 ± 5.66	72.22 ± 5.95

**Table 8:** Confusion matrix of the classification from the worst results produced by ConVNet and SVM-GA.

Class	Lymphocyte	Pre-T	Pre-B
*ConVNet*			
Lymphocyte	**31**	0	0
Pre-T	0	**28**	17
Pre-B	0	9	**36**

*SVM-GA*			
Lymphocyte	**28**	0	3
Pre-T	0	**27**	18
Pre-B	0	4	**41**

**Table 9:** Confusion matrix of the classification from the best results produced by ConVNet and SVM-GA.

Class	Lymphocyte	Pre-T	Pre-B
*ConVNet*			
Lymphocyte	**36**	0	0
Pre-T	0	**37**	8
Pre-B	0	9	**36**

*SVM-GA*			
Lymphocyte	**30**	1	0
Pre-T	0	**38**	7
Pre-B	0	8	**37**

## Data Availability

The microscopic images are acquired from two different collections [6, 7]. The images have been rescaled to equal size of 256 × 256 pixels, and each contains a single cell. The image data used in this work are available at http://mcs.sat.psu.ac.th/dataset/dataset.zip.

## Additional Points

*Hardware and Software.* The computer used for our experiments is a Mac Pro configured with 3.7 GHz, Quad-Core Intel Xeon E5, 12 GB DDR3 and GPU AMD FirePro D300 2048 MB, running Mac OS X 10.11.6. The implementation is with python 2.7.12, Keras 1.2.2, and scikit-learn 0.19.2.
